# Immunological landscape of periodontitis and rheumatoid arthritis and their molecular crosstalk

**DOI:** 10.1186/s40001-025-02376-y

**Published:** 2025-02-22

**Authors:** Weimin Zhao, Chenxu Liu, Xiangzhi Cui, Qianjiang Chen

**Affiliations:** 1 The Seventh People’s Hospital of Zhengzhou City, Zhengzhou, China; 2 School of Basic Medical Science, Zhengzhou University, Zhengzhou, China; 3 The First Affiliated Hospital of Zhengzhou University, Zhengzhou, China

**Keywords:** Periodontitis, Rheumatoid arthritis, PTPRC, Molecular crosstalk

## Abstract

**Background:**

The association between periodontitis (PT) and rheumatoid arthritis (RA) is well-established; however, the molecular mechanisms underlying this relationship remain poorly understood. This study aims to delineate shared genetic and molecular features between PT and RA to uncover potential common pathways involved in their pathogenesis.

**Methods:**

Gene expression data sets for PT and RA were retrieved from the Gene Expression Omnibus (GEO) database. Differentially expressed genes (DEGs) and co-expressed gene modules were identified using weighted gene co-expression network analysis (WGCNA) and the DESeq2 package. Enrichment analyses, including KEGG and Gene Ontology (GO) pathways, as well as immune cell infiltration profiling, were performed to explore shared biological pathways. A protein–protein interaction (PPI) network was constructed to pinpoint key genes linking PT and RA. Functional assays were conducted by overexpressing the identified core gene, PTPRC, in MH7A cells via lentiviral transfection, followed by cell viability (CCK-8), migration, and invasion assays. In addition, transcription factor enrichment and connectivity map (cMAP) analyses were employed to identify common transcriptional regulators and potential therapeutic targets for both conditions.

**Results:**

WGCNA and DESeq2 analyses revealed 154 shared DEGs between PT and RA, predominantly enriched in immune and inflammatory response pathways. PTPRC emerged as a pivotal shared gene, exhibiting significantly higher expression in PT patients compared to controls. In vitro assays confirmed that PTPRC overexpression enhanced fibroblast proliferation, migration, and invasion. Furthermore, transcription factor enrichment analysis and cMAP identified overlapping transcriptional regulators and potential pharmacological agents for both diseases.

**Conclusions:**

This study provides novel insights into shared gene expression profiles and molecular mechanisms linking PT and RA, identifying PTPRC as a potential key regulator. These findings suggest that targeting PTPRC could offer therapeutic opportunities for RA driven by PT.

## Introduction

Periodontitis (PT) ranks as the sixth most prevalent chronic inflammatory disease globally, affecting approximately 743 million individuals, with severe cases accounting for nearly 11% of the population [1–3]. PT is characterized by the progressive destruction of periodontal tissues, including alveolar bone, gingiva, periodontal ligament, and cementum, predominantly driven by bacterial biofilms [4, 5]. These biofilms provoke an excessive pro-inflammatory response within the gingival connective tissues, resulting in paracrine signaling and eventual alveolar bone resorption [6]. The progressive degradation of bone culminates in tooth mobility and loss, severely impairing quality of life and imposing substantial social and economic burdens [7, 8]. Moreover, PT has been strongly associated with various systemic conditions, including rheumatoid arthritis (RA), infective endocarditis, and Alzheimer’s disease [9–11].

RA is a prevalent autoimmune disease (AID) with a global incidence of approximately 1% [12, 13]. It is primarily characterized by immune system dysregulation, synovitis, and progressive bone and cartilage degradation [13]. Despite extensive research, the precise pathogenesis of RA remains elusive, complicating the development of effective therapeutic interventions [14]. Current RA treatments, including disease-modifying antirheumatic drugs (DMARDs), are often limited by adverse effects, suboptimal response rates, and the emergence of drug resistance. Thus, elucidating the molecular mechanisms underlying RA pathogenesis is critical for advancing therapeutic strategies.

Emerging evidence suggests that PT plays a contributory role in the onset and progression of RA, particularly in anti-citrullinated protein antibody (ACPA)-positive RA [15, 16]. Non-surgical periodontal therapy has been shown to reduce systemic inflammatory markers, potentially mitigating RA severity [17]. In addition, several periodontal pathogens have been implicated in the initiation and exacerbation of RA [18, 19].

However, the molecular pathways linking PT and RA remain poorly understood. In this study, we employed bioinformatics analyses to investigate gene expression data sets related to PT and RA, sourced from the GEO database. Our aim was to identify shared pathogenic genes, immunological features, and molecular mechanisms that bridge these two diseases. In addition, we explored transcription factors and potential therapeutic targets, further validating a core pathogenic gene through in vitro experiments.

## Materials and methods

### Data source

The gene expression omnibus (GEO; http://www.ncbi.nlm.nih.gov/geo) is a publicly accessible repository that hosts a comprehensive collection of high-throughput sequencing and microarray data sets submitted by research groups worldwide. To identify relevant data sets, we conducted a keyword search for “rheumatoid arthritis” and “periodontitis.” Based on this search, we selected three microarray data sets: GSE223924, GSE206848, and GSE48780.

The GSE223924 data set consists of gene expression profiles from 10 healthy individuals and 10 patients diagnosed with periodontitis. The GSE206848 data set includes synovial tissue samples from 7 healthy individuals and 2 rheumatoid arthritis patients. Finally, the GSE48780 data set comprises 83 synovial samples from rheumatoid arthritis patients. Both RA data sets (GSE206848 and GSE48780) were generated using the GPL570 [HG-U133_Plus_2] Affymetrix Human Genome U133 Plus 2.0 Array platform.

### Identification of differentially expressed genes (DEGs)

The selected data sets were accessed using the R package GEOquery. To identify differentially expressed genes (DEGs) in each data set, we applied the DESeq2 package in R, with significance thresholds set at a *p* < 0.05 and an absolute log2 fold change (|log2 FC|) > 1. DEGs were visualized using volcano plots and heatmaps, which were generated using the R package ggplot2.

### Construction of a weighted gene co-expression network to identify key genes associated with PT and RA

Weighted gene co-expression network analysis (WGCNA) was utilized to identify potential gene interactions and associations with disease phenotypes by analyzing gene co-expression relationships across samples. This approach is particularly valuable for investigating complex relationships between gene expression profiles and phenotypic traits. Co-expression analysis of the PT data set (GSE223924) and RA data sets (GSE206848, GSE48780) was performed using the Wekemo Bioincloud platform (https://www.bioincloud.tech) to construct a weighted gene co-expression network [20]. Soft-thresholding power selection: To determine the optimal soft-thresholding power, we evaluated the scale-free topology fit index across a range of powers (1–20). The power that achieved an *R*^*2*^ value greater than 0.85 was selected, as it indicated a scale-free network topology while balancing network connectivity and sparsity effectively.

Module identification parameters: Using the optimal soft-thresholding power, gene modules were constructed with the blockwiseModules function in R. The following parameters were applied for module identification: minimum module size: 30 genes, ensuring robust module formation. Dynamic tree cut method: Hybrid dynamic tree cutting with a deepSplit parameter of 2 for fine-grained module detection. Module merging threshold: Modules with eigengene correlations above 0.25 were merged to reduce redundancy and enhance biological relevance.

To visualize these associations, a module–disease correlation heatmap was generated using R software. The module with the highest correlation to the disease was selected as the key module. Genes exhibiting a gene significance (GS) score > 0.5 and a *p* < 0.05 were considered strongly associated with both the disease and the module. These genes were subsequently chosen as candidate genes for further analysis.

### Enrichment analyses of DEGs

To functionally annotate and visualize the differentially expressed genes (DEGs), the Metascape database (www.metascape.org) was employed [21]. Gene Ontology (GO) and Kyoto Encyclopedia of Genes and Genomes (KEGG) pathway enrichment analyses were conducted to identify the biological processes and signaling pathways implicated in the pathogenesis and progression of both diseases. Enrichment was deemed statistically significant when the overlap between gene sets was > 3, with a *p* < 0.01.

### Integration and analysis of protein–protein interactions

The protein–protein interaction (PPI) network was constructed by integrating the DEGs using the STRING database (www.string-db.org), with a median confidence score threshold of 0.4. The resulting PPI network was then visualized and further analyzed using Cytoscape software.

### Immune infiltration analysis

Immune cell infiltration was assessed using the “CIBERSORT” package, which estimates the abundance and proportion of various immune cell types based on gene expression profiles. The results were visualized as bar plots, generated with the “ggplot2” package to illustrate the distribution of immune cell types in each sample.

### Cell type signatures and transcription factor targets enrichment analysis

The Metascape database (www.metascape.org) was utilized to conduct analyses of Transcription Factor Targets (TFT) and Cell Type Signatures [21]. Co-expressed genes identified in both periodontitis and rheumatoid arthritis data sets were input into the database for enrichment analysis. The results were visualized using bar graphs to represent the significance of various cell type signatures and transcription factor targets.

### Connectivity map (cMAP) analysis

The Connectivity Map (cMAP; https://clue.io) is a comprehensive database designed to elucidate relationships between gene expression profiles, diseases, and small molecule compounds. In this study, upregulated and downregulated co-expressed genes were queried in the cMAP database to identify potential small-molecule drugs for treating periodontitis and rheumatoid arthritis. The top 10 compounds with the highest enrichment scores were selected for further evaluation as potential therapeutic agents.

### Human periodontal tissue collection

Human periodontal tissues were collected from patients at the Department of Stomatology, Zhengzhou Seventh People’s Hospital. Samples included healthy teeth and teeth from patients with periodontitis, obtained during routine tooth extractions. This study was approved by the Ethics Committee of Zhengzhou Seventh People’s Hospital, and informed consent was obtained from all participants prior to the collection of samples.

### Cell culture

Fibroblast-like synoviocytes from rheumatoid arthritis patients (MH7A cells; Riken Cell Bank, Ibaraki, Japan), with passage numbers between four and eight, were utilized for experimental validation. The cells were cultured in high-glucose DMEM medium (Hyclone, USA) supplemented with 20% heat-inactivated fetal bovine serum (FBS; Millipore, USA), 100 U/ml of penicillin, and 100 µg/ml of streptomycin (both from Beyotime, China). Cultures were maintained at 37 °C in a humidified atmosphere with 5% CO_2_.

### Construction of MH7A cells overexpressing PTPRC

A PTPRC expression construct was synthesized and inserted into the recombinant lentiviral vector GV358 (Shanghai GeneChem Co., Ltd.). The recombinant lentiviral vector was used to achieve PTPRC overexpression, with an empty vector serving as a control. MH7A cells were seeded into 96-well plates at a density of 5 × 10^3^ cells per well in 100 µl of complete medium. The cells were infected with three different multiplicities of infection (MOI = 10, MOI = 20, MOI = 30) using the FuGENE® 6 transfection reagent (Nanjing KeyGen Biotech Co., Ltd.). After transfection, the cells were incubated for 24 and 48 h before further experiments.

### Western blot

Total protein was extracted from the cells/tissues using lysis buffer, and protein concentration was determined using the BCA Protein Assay Kit (Thermo Fisher Scientific). Equal amounts of protein (30 µg per lane) were separated by 10% sodium dodecyl sulfate–polyacrylamide gel electrophoresis (SDS–PAGE) and transferred to polyvinylidene fluoride (PVDF) membranes (Millipore, IPVH00010) using the wet transfer method at 100 V for 1 h. The PVDF membranes were blocked with 5% skim milk in tris-buffered saline with 0.1% Tween 20 (TBST) for 1 h at room temperature to prevent non-specific binding. Membranes were then incubated overnight at 4 °C with the following primary antibodies: PTPRC antibody (Abcam, ab317446), diluted at 1:1000. β-Actin antibody (Proteintech Group, HRP-66009), diluted at 1:3000. After three washes with TBST, membranes were incubated with HRP-conjugated secondary antibodies for 1 h at room temperature. Following the secondary incubation, the protein bands were visualized using SuperSignal™ West Pico PLUS Chemiluminescent Substrate (Thermo Fisher Scientific) according to the manufacturer’s instructions. The chemiluminescence signal was detected using a Gel Imaging System (Tanon Science & Technology Co., Shanghai, China). Protein levels were quantified using ImageJ software and normalized to the control (β-actin).

### Transwell migration and invasion assay

Migration and invasion assays were conducted using transwell chambers (Millipore, Billerica, USA). For the migration assay, transfected cells (2.5 × 10^4^ cells) were seeded into the upper chamber in serum-free medium, while the lower chamber contained DMEM medium supplemented with 10% FBS. In the invasion assay, the upper chamber was pre-coated with Matrigel (BD Biosciences, Franklin Lakes, NJ, USA), and the remaining procedure mirrored that of the migration assay. After 24 h of incubation, cells that migrated or invaded through the membrane were fixed and stained with crystal violet. The number of migrated or invaded MH7A cells was quantified by counting cells in 3 random fields under an inverted light microscope at 200 × magnification.

### Cell counting kit-8 (CCK-8) assay

To assess cell proliferation, MH7A cells were seeded into 96-well plates at a density of 2000 cells per well and cultured for the specified durations. Cell viability was measured using the cell counting kit-8 (CCK-8) solution according to the manufacturer’s instructions (A311-01, Vazyme Biotech). Absorbance at 450 nm was determined using a microplate reader.

### Statistical analysis

All experiments were performed at least three times to ensure reproducibility, and the data are presented as the mean ± standard deviation (SD). Statistical analysis was performed using GraphPad Prism 9.0 and SPSS 27.0 software. For comparisons between two groups, the Student’s *t* test was used when data followed a normal distribution. For non-normally distributed data, the Mann–Whitney *U* test was applied. For multiple comparisons between more than two groups, a one-way analysis of variance (ANOVA) was conducted, followed by post-hoc Tukey’s test for pairwise comparisons. Pearson’s correlation coefficient was used for correlation analysis of normally distributed data. A *p* < 0.05 was considered statistically significant.

## Results

### Identification of differentially expressed genes in periodontitis and rheumatoid arthritis

**Fig. 1:**
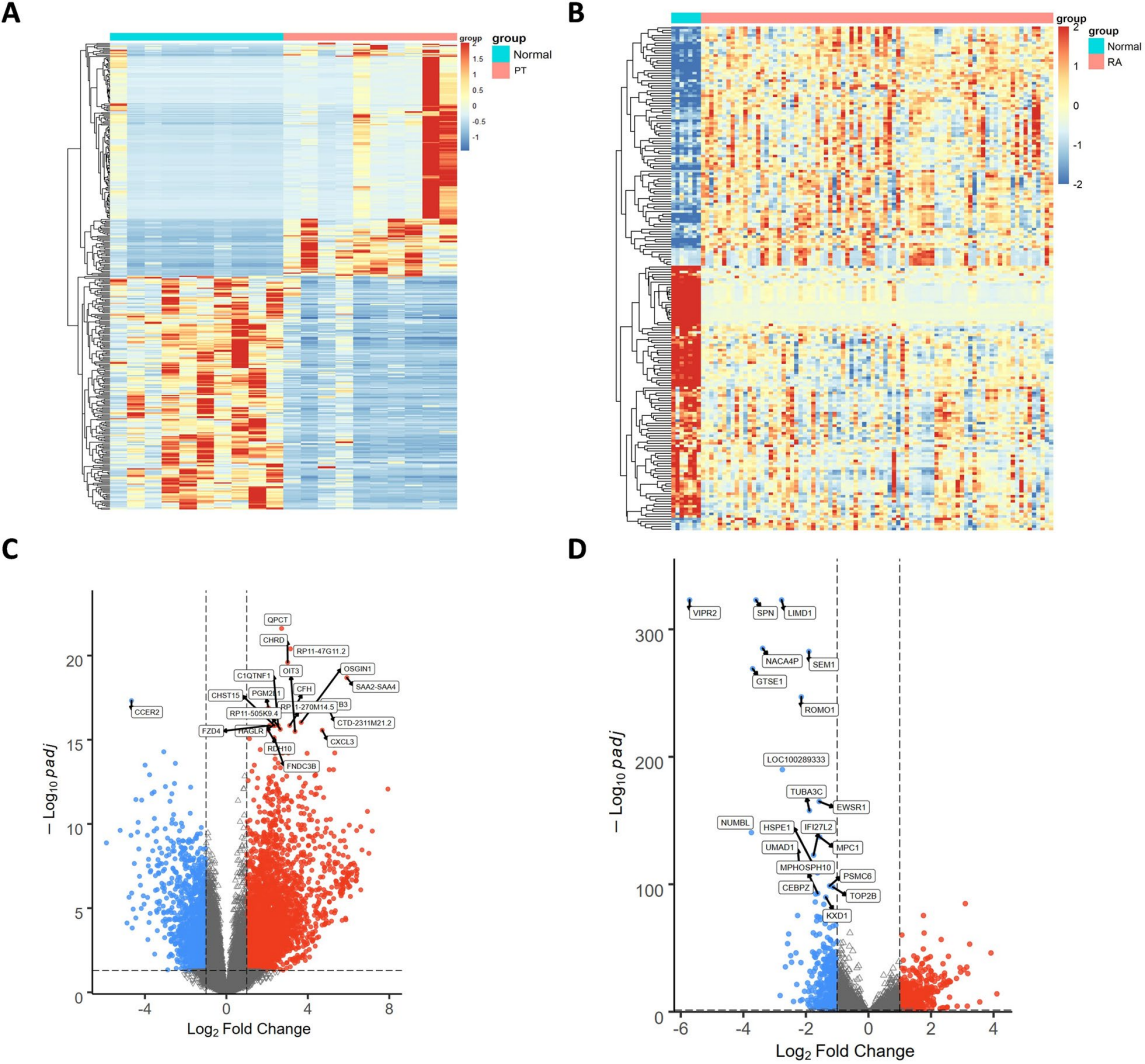
Heatmaps and volcano maps. **A** Heatmaps of PT (GSE223924). **B** Heatmaps of RA (GSE206848, GSE48780). **C** Volcano map of PT (GSE223924). **C** Volcano map of RA (GSE206848, GSE48780). Upregulated genes are marked in light red; downregulated genes are marked in light blue

From the periodontitis (PT) data set (GSE223924), we identified a total of 4,633 differentially expressed genes (DEGs). A heatmap was generated to visualize the top 200 genes with the most pronounced upregulation and downregulation (Fig. 1A). The volcano plot depicted 3396 upregulated and 1237 downregulated DEGs (Fig. 1C). In the rheumatoid arthritis (RA) data sets (GSE206848, GSE48780), we identified 1,214 DEGs, including 784 upregulated and 430 downregulated genes (Fig. 1B, D).

### Weighted gene co-expression network analysis of periodontitis and rheumatoid arthritis

**Fig. 2:**
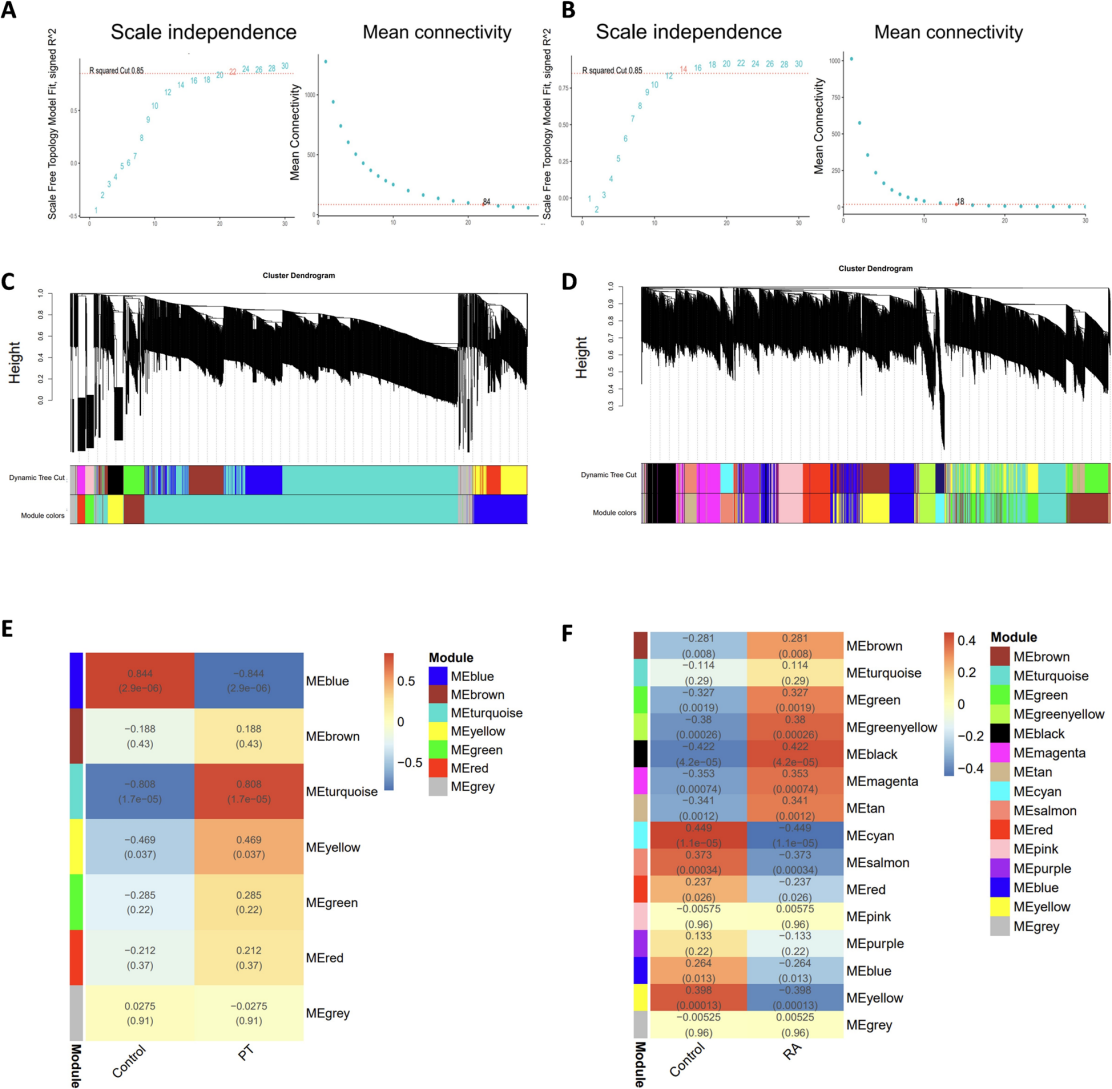
Identification of modules linked to clinical features of PT and RA using WGCNA. **A** Determination of soft-threshold power for PT. **B** Determination of soft-threshold power for RA. **C** Cluster dendrogram of co-expressed genes in PT. **D** Cluster dendrogram of co-expressed genes in RA. **E** Heatmap of module–trait relationships in PT. **F** Heatmap of module–trait relationships in RA

We conducted weighted gene co-expression network analysis (WGCNA) to investigate the correlation between gene expression profiles and clinical traits for both PT and RA data sets. For the PT data set (GSE223924), a soft-thresholding power of 22 was chosen based on scale independence and mean connectivity, resulting in the identification of seven modules (Fig. 2C). A module–trait relationship heatmap, constructed using Spearman's correlation coefficients, revealed that the turquoise module exhibited the strongest positive correlation with PT (*r* = 0.808, *p* = 1.7 × 10⁻^5^), while the blue module showed the strongest negative correlation (*r* = − 0.844, *p* = 2.9 × 10⁻⁶) (Fig. 2E).

Similarly, for the RA data sets (GSE206848, GSE48780), a soft-thresholding power of 14 was selected based on scale independence and mean connectivity, leading to the identification of 15 modules (Fig. 2D). Among these, the black module exhibited the strongest positive correlation with RA (*r* = 0.422, *p* = 4.2 × 10⁻^5^), whereas the cyan module demonstrated the strongest negative correlation (*r* = − 0.449, *p* = 1.1 × 10⁻^5^).

### GO and KEGG pathway enrichment analysis

**Fig. 3:**
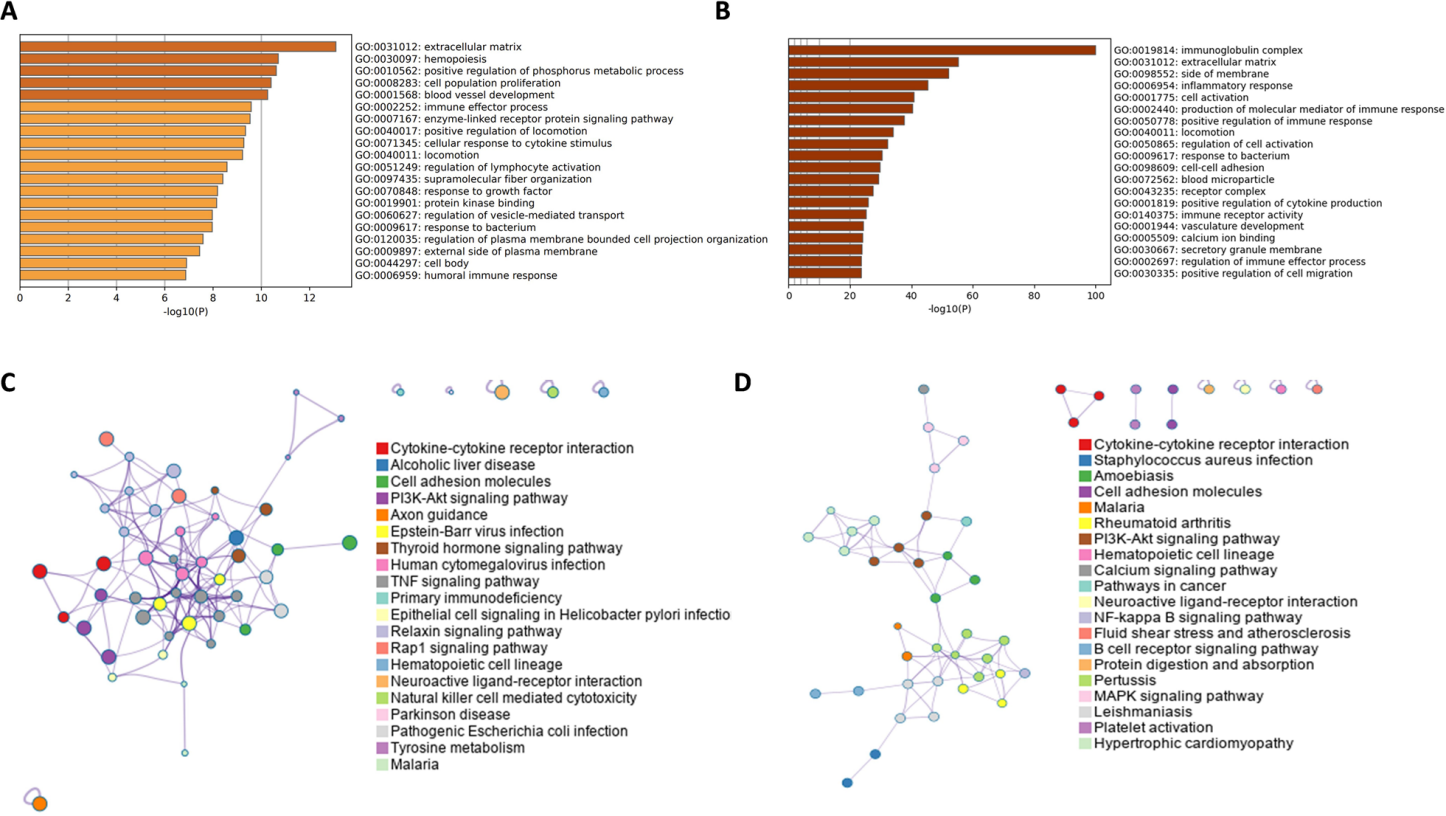
GO and KEGG functional enrichment analyses identify key biological processes and pathways of differentially expressed genes in PT and RA data sets. **A** GO analysis of DEGs in the PT data set. **B** GO analysis of DEGs in the RA data set. **C** KEGG pathway enrichment of DEGs in the PT data set. **D** KEGG pathway enrichment of DEGs in the RA data set

Gene Ontology (GO) and Kyoto Encyclopedia of Genes and Genomes (KEGG) pathway enrichment analyses were performed on differentially expressed genes (DEGs) from the periodontitis (PT) and rheumatoid arthritis (RA) data sets using the DESeq2 package. GO analysis of the PT data set revealed significant enrichment in pathways associated with the extracellular matrix, hematopoiesis, phosphate metabolism regulation, and immune responses (Fig. 3A). KEGG analysis identified significant involvement of PT DEGs in pathways related to cytokine signaling, cell adhesion molecules, tumor necrosis factor (TNF) signaling, PI3K–AKT signaling, thyroid hormone synthesis, bacterial infections, and certain viral infections (Fig. 3C).

For RA-related DEGs, GO enrichment analysis similarly indicated significant associations with extracellular matrix processes, bacterial response, and immune-related pathways (Fig. 3B). KEGG pathway analysis highlighted significant enrichment in pathways involving cytokine–cytokine receptor interactions, neuroactive ligand–receptor interactions, rheumatoid arthritis-specific B cell signaling, MAPK signaling, NF-κB signaling, and PI3K–AKT signaling (Fig. 3D).

### Protein–protein interaction network and immune cell infiltration analysis

**Fig. 4:**
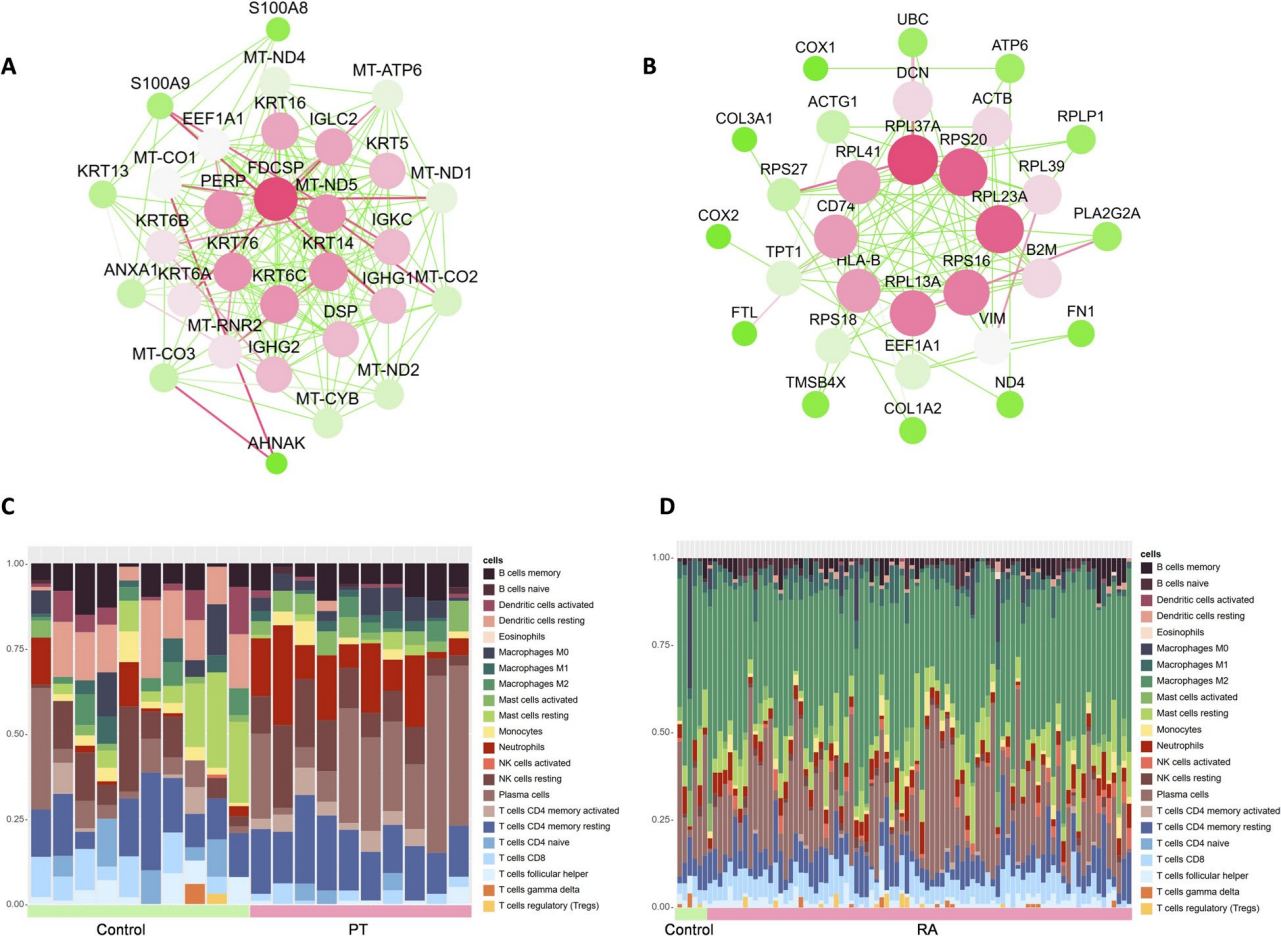
Correlation of hub genes and immune cell infiltration in PT and RA data sets. **A** PPI network of DEGs in the PT data set. **B** PPI network of DEGs in the RA data set. **C** Heat map of the relative proportions of 22 types of infiltrating immune cells in patients with PT. **D** Heat map of the relative proportions of 22 types of infiltrating immune cells in patients with RA

Protein–protein interaction (PPI) networks for DEGs in the PT data set (GSE223924) were constructed using the STRING database (Fig. 4A). The top 10 genes with the highest interaction scores included FDCSP, MT-ND5, KRT14, KRT6C, KRT76, PERP, KRT16, IGLC2, KRT5, and IGKC. For the RA data sets (GSE206848 and GSE48780), PPI analysis identified the top 10 interacting genes as RPL37A, RPS20, RPL23A, RPS16, RPL13A, HLA-B, CD74, RPL41, DCN, and ACTB (Fig. 4B).

In addition, immune cell infiltration analysis was conducted for both disease data sets. In the PT data set, 22 types of immune cells were identified, with notable increases in the infiltration of naïve B cells, plasma cells, activated mast cells, and neutrophils in periodontitis patients. In the RA data set, there was a slight increase in the infiltration of naïve B cells, CD8 + T cells, activated CD4 + memory T cells, regulatory T cells (Tregs), and M0 macrophages in rheumatoid arthritis patients.

### Analysis of shared genes and functional enrichment

**Fig. 5:**
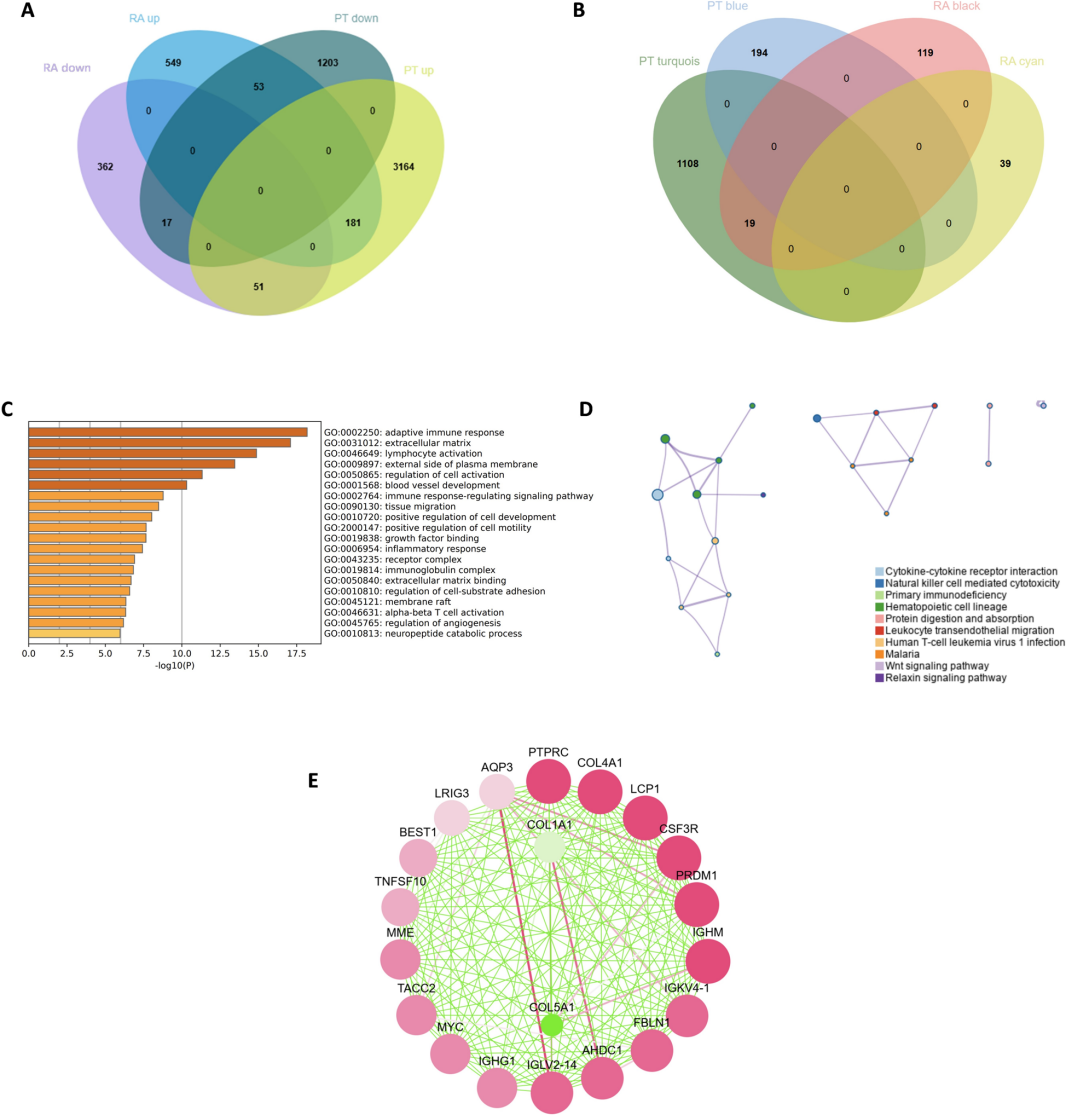
Functional and pathway enrichment analysis of shared genes between PT and RA. **A** Venn diagram showing common up-regulated and down-regulated differentially expressed genes in PT and RA data sets. **B** Venn diagram illustrating the shared genes between two PT modules and two RA modules. **C** GO enrichment analysis of shared genes between PT and RA data sets. **D** KEGG pathway enrichment analysis of shared genes between PT and RA data sets. **E** PPI network of shared genes between PT and RA data sets

To elucidate the shared pathogenic mechanisms between periodontitis (PT) and rheumatoid arthritis (RA), we analyzed the intersection of differentially expressed genes (DEGs) and genes selected through weighted gene co-expression network analysis (WGCNA) from both data sets. We identified 198 DEGs common to both PT and RA, showing consistent expression trends in both diseases (Fig. 5A). WGCNA revealed 19 overlapping genes between PT and RA (Fig. 5B), which we hypothesize are involved in the pathogenesis of both conditions and may play a shared role.

Functional annotation and enrichment analysis of these shared genes were performed to investigate the potential biological changes common to PT and RA (Fig. 5C, D). Gene Ontology (GO) analysis of the shared genes indicated significant enrichment in immune and inflammatory processes, including lymphocyte activation, inflammatory response, adaptive immune response, immunoglobulin-related pathways, and immune response regulation. Kyoto encyclopedia of genes and genomes (KEGG) analysis highlighted significant involvement in cytokine signaling and immune-related pathways, such as cytokine–cytokine receptor interaction, natural killer cell-mediated cytotoxicity, and primary immune response. Protein–protein interaction (PPI) analysis of these intersecting genes identified PTPRC as the top-ranked gene by interaction degree, suggesting its pivotal role as a bridging factor in both diseases.

### The key role of PTPRC in periodontitis and rheumatoid arthritis

**Fig. 6:**
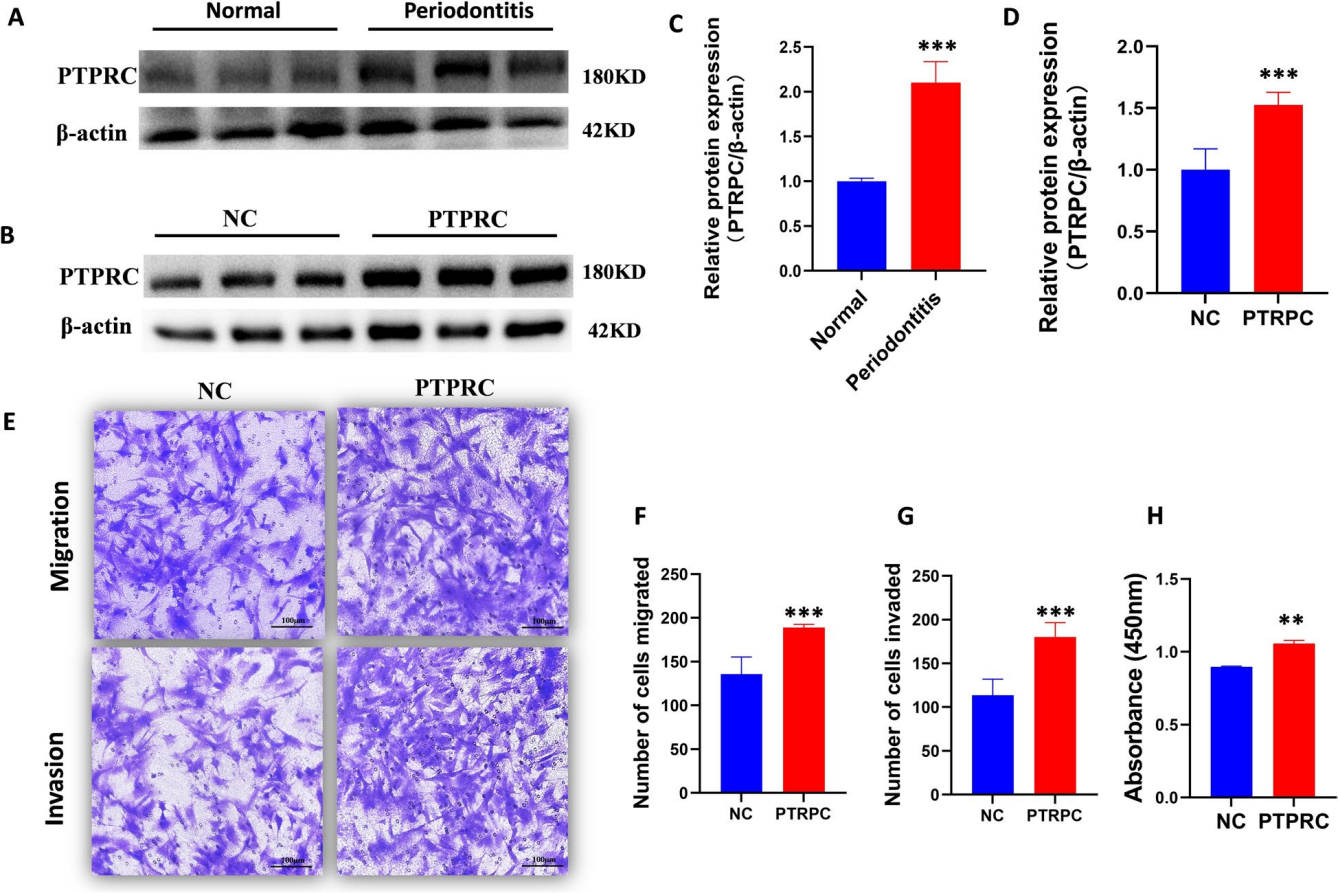
Expression of PTPRC in periodontal tissues and its effects on MH7A cells. **A** Protein expression of PTPRC in periodontal tissues (*n* = 3). **B** Overexpression of PTPRC protein in MH7A cells after plasmid transfection for PTPRC overexpression (*n* = 3). **C** Quantitative analysis of PTPRC protein expression in periodontal tissues. **D** Quantitative analysis of PTPRC protein expression in MH7A cells. **E** Effects of PTPRC overexpression on the migration and invasion capabilities of MH7A cells. **F**, **G** Quantitative analysis of migration (*n* = 3) and invasion (*n* = 3) assays in MH7A cells. **H** Effect of PTPRC overexpression on the proliferative capacity of MH7A cells assessed by CCK-8 assay (*n* = 5). Data were analyzed using Student’s *t* test, **P < 0.01 and ***P < 0.001 vs. NC

To validate the role of PTPRC in periodontitis, we analyzed periodontal tissue samples from patients with periodontitis and healthy controls. Western blot analysis demonstrated a significant increase in PTPRC expression in periodontal tissues from patients with periodontitis compared to controls (Fig. 6A, C). This finding supports the hypothesis that elevated PTPRC may contribute to the onset and exacerbation of rheumatoid arthritis. We further investigated the effects of PTPRC overexpression on MH7A cells by designing primers for its high expression and generating PTPRC-overexpressing plasmid constructs (Fig. 6B, D). Transfected MH7A cells were assessed for migration, invasion, and proliferation capabilities. Results showed that PTPRC overexpression significantly enhanced the migration, invasion, and proliferation of MH7A cells (Fig. 6E–H), indicating its potential role in driving disease progression.

Each experimental condition was performed in three biological replicates to ensure robust and reproducible results. For the migration and invasion assays, after the completion of the experiments, three random fields were photographed per well under a microscope, and the number of cells in each field was manually counted.

### Additional analyses of shared genes: transcription factor targets, cell type signatures, and potential therapeutic agents

**Fig. 7:**
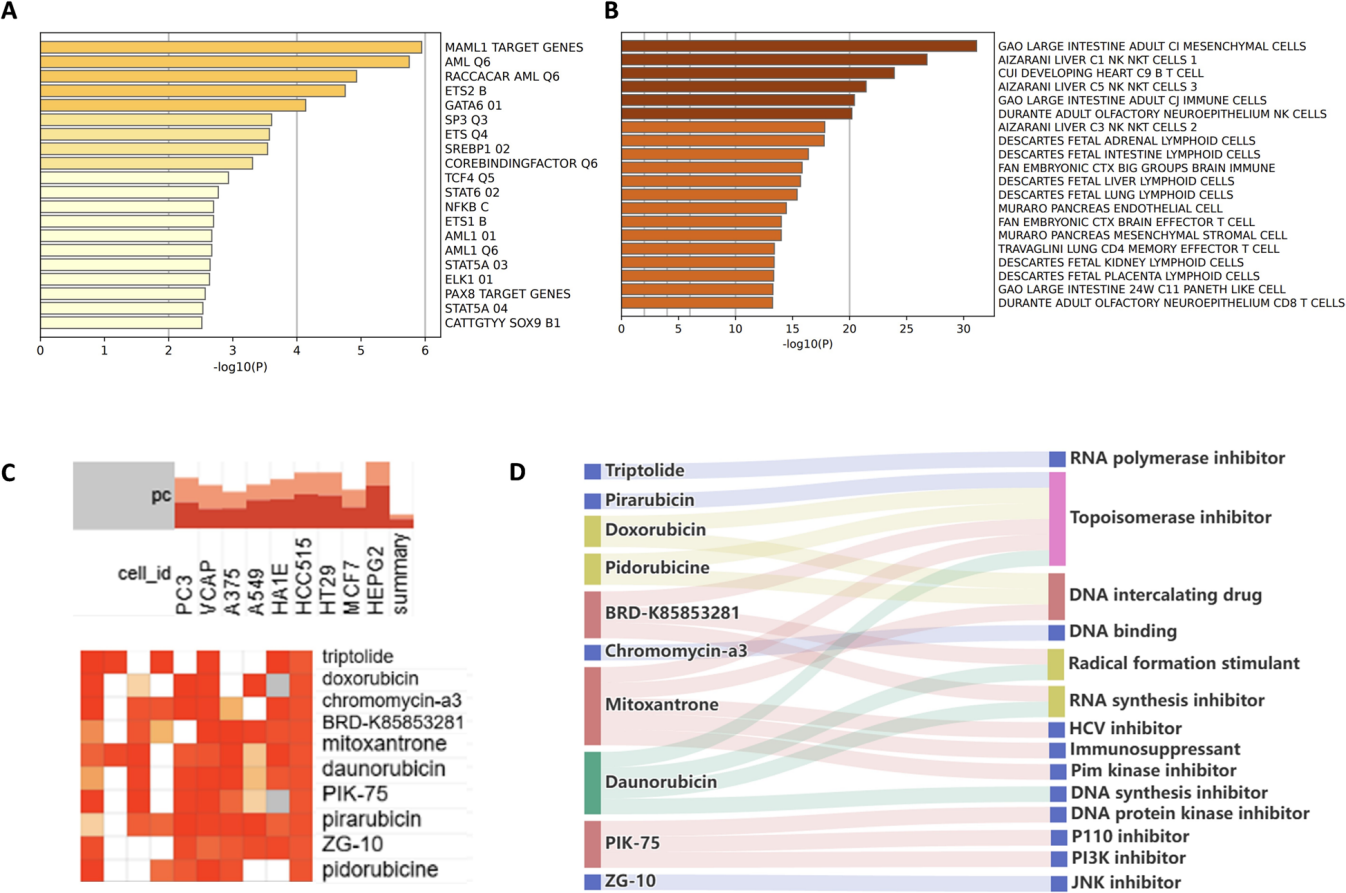
Analyze shared genes in terms of transcription factor targets, cell type signatures, and potential therapeutic agents. **A** Transcription factor target analysis of shared genes between PT and RA data sets. **B** Cell type signature analysis of shared genes between PT and RA data sets. **C** Identification of potential therapeutic compounds targeting shared genes using the cMAP database. **D** Classification of the top 10 potential therapeutic compounds

To identify potential transcription factors involved in regulating the shared genes between PT and RA, we performed Transcription Factor Targets analysis, revealing several transcription factors that may be crucial in the regulation of these genes (Fig. 7A). Cell Type Signatures analysis further characterized the expression patterns of these genes across different cell types (Fig. 7B).

In addition, we explored potential small-molecule drugs for therapeutic applications in RA patients related to PT by analyzing upregulated and downregulated shared genes using the Connectivity Map (cMAP) database. The top 10 compounds with the highest enrichment scores were triptolide, doxorubicin, chromomycin-A3, BRD-K85853281, mitoxantrone, daunorubicin, PIK-75, pirarubicin, ZG-10, and pidorubicin (Fig. 7C). The targeted pathways and classifications of these compounds are detailed in Fig. 7D.

## Discussion

Rheumatoid arthritis (RA) is a chronic autoimmune disorder affecting approximately 1% of the global population, with a significantly higher incidence in compared to men [12, 22]. Although RA can occur at any age, it is most commonly diagnosed between the ages of 30 and 60 [23]. The disease is characterized by persistent joint pain, stiffness, and swelling, which, if left untreated, can lead to severe joint deformities and loss of function [24, 25]. These symptoms often cause substantial mobility issues, limiting daily activities and reducing work productivity. RA can also have systemic effects, such as fatigue, weight loss, fever, and the involvement of internal organs, such as the heart and lungs, increasing the risk of cardiovascular complications [26, 27]. In addition, the chronic pain and functional limitations associated with RA can lead to depression and anxiety, further diminishing the quality of life [28, 29].

The pathogenesis of RA is complex, but evidence suggests a significant relationship between RA and periodontitis [30]. Both conditions are characterized by abnormal immune responses and chronic inflammation [31]. Common immune markers, such as rheumatoid factor and anti-citrullinated protein antibodies, are present in both periodontitis and RA, indicating shared pathophysiological mechanisms [32–34]. Systemic inflammatory mediators, including tumor necrosis factor-alpha (TNF-α) and interleukins, induced by periodontitis, may exacerbate RA by influencing systemic inflammation and joint involvement [16, 35]. Epidemiological studies have noted a higher incidence of RA in individuals with periodontitis, and treatment of periodontitis has occasionally alleviated RA symptoms, suggesting a potential systemic inflammatory link [36, 37]. Shared risk factors, including smoking and genetic predispositions, may also contribute to the susceptibility of both conditions [38].

In this study, we employed weighted gene co-expression network analysis (WGCNA) on GEO data sets for RA and periodontitis to identify potential shared genes. By analyzing gene intersections from relevant modules and performing differential gene analysis using the DESeq2 package, we identified overlapping genes between the two conditions. Enrichment analysis of these shared genes revealed their involvement in critical pathways, such as cytokine–cytokine receptor interactions, cell adhesion molecules, the PI3K–AKT signaling pathway, extracellular matrix dynamics, and immune-related cellular processes. These pathways may be pivotal in the development and progression of both diseases.

Our subsequent enrichment and protein–protein interaction (PPI) analyses identified PTPRC as a core target gene. PTPRC, also known as CD45, is a transmembrane glycoprotein expressed on nearly all hematopoietic cells, excluding mature erythrocytes [39, 40]. It plays a crucial role in T and B cell antigen receptor-mediated activation and is composed of two cytoplasmic domains, a transmembrane domain, and an extracellular domain, representing approximately 10% of surface antigens on positively expressing cells [39]. PTPRC’s function in modulating antigen receptor signaling is vital for both adaptive immunity and the maintenance of chronic inflammatory states [41]. Although this study observed significantly increased PTPRC expression in periodontitis tissues, it remains to be determined whether this overexpression directly influences RA onset or exacerbation. Existing literature suggests that elevated CD45 levels are associated with hyperactive immune responses and increased pro-inflammatory cytokine production, both of which are known contributors to RA progression [42, 43].

In the context of PT-driven inflammation, local immune activation resulting from periodontal disease may facilitate the migration, activation, and proliferation of immune cells via PTPRC, thereby exacerbating the inflammatory milieu characteristic of RA. Specifically, the persistent inflammatory response in periodontitis leads to the activation of immune cells, which secrete a wide array of pro-inflammatory cytokines, such as TNF-α, IL-1β, and IL-6. These cytokines not only act locally but also disseminate systemically through the bloodstream, amplifying the inflammatory response and contributing to the worsening of RA. In this regard, PTPRC, a pivotal regulator of immune cell signaling, enhances the activation and responsiveness of T and B cells, potentiating their reaction to pro-inflammatory mediators and further amplifying systemic immune dysregulation, ultimately driving RA progression.

Moreover, PTPRC may also exacerbate local inflammation by promoting immune cell migration and infiltration, particularly within the synovial joints. The accumulation of immune cells within the joint space leads to the release of additional inflammatory cytokines, further contributing to joint damage and bone erosion—hallmarks of RA pathophysiology. In addition, dysregulation of immune tolerance mediated by PTPRC may result in aberrant immune responses against self-tissues, propelling the pathogenesis of RA. Collectively, PTPRC serves as a critical mediator within the inflammatory framework induced by PT, providing a molecular link between periodontitis and RA through its modulation of immune responses across multiple interconnected pathways.

We used the cMAP database to explore potential drug candidates and identified several molecules, such as triptolide and doxorubicin, which could modulate the shared genetic framework of periodontitis and RA. These findings suggest that developing targeted therapies capable of addressing both conditions simultaneously could offer a novel approach to managing these interconnected diseases. The cMAP database consists of a curated collection of gene expression profiles from various cell lines treated with a wide range of compounds [44]. However, it is important to note that the database may not include all available compounds, particularly novel ones or those not widely studied [45]. In addition, the cMAP database relies on specific cell line models, meaning that results may not fully represent the diversity of responses seen in different disease contexts or patient populations.

This study heavily relies on publicly available gene expression data sets, such as those from gene expression omnibus (GEO) and other repositories. While these data sets offer valuable insights and large sample sizes, they may not fully represent the diversity of the population or the heterogeneity of disease conditions. In addition, the data in these repositories may be subject to batch effects or inconsistencies across different studies, which can affect the generalizability of our findings.

This study is observational in nature, meaning that while we have identified potential biomarkers and therapeutic targets, causality cannot be definitively established from our analysis alone. The observed associations between PTPRC and disease progression in periodontitis and rheumatoid arthritis need further validation in experimental models, such as animal studies or clinical trials, to confirm causality.

Given PTPRC's critical role in regulating immune cell activation and inflammation, targeting it could represent a promising therapeutic approach for diseases, such as rheumatoid arthritis (RA) and periodontitis. Inhibiting PTPRC may help suppress excessive immune responses, potentially mitigating the tissue damage seen in these conditions. Small molecules or monoclonal antibodies targeting PTPRC could be developed to modulate immune cell signaling, providing a novel method to reduce inflammation and improve clinical outcomes.

Furthermore, PTPRC may serve as a valuable biomarker for the early detection and monitoring of RA and periodontitis. Elevated expression of PTPRC on immune cells may signal ongoing immune activation, facilitating earlier interventions and more personalized treatment strategies. Its use as a biomarker could also help track disease progression and assess the efficacy of targeted therapies, making it an important tool for both diagnosis and treatment monitoring.

In conclusion, this study highlights the intricate molecular crosstalk between periodontitis and RA, identifying PTPRC as a potential key regulator in both diseases. Our findings enhance the understanding of shared immunological mechanisms and open new avenues for developing dual-targeted therapies to improve outcomes for patients suffering from these chronic inflammatory disorders.

## Data Availability

Data is provided within the manuscript or supplementary information files.
